# ‘Two countries-two labs’: the transnational gamete donation (TGD) programme to support egg donation

**DOI:** 10.1007/s10815-020-01961-w

**Published:** 2020-10-12

**Authors:** Maria Elisabetta Coccia, Francesca Rizzello, Savio Wakunga, Laura Badolato, Paolo Evangelisti, Francesco Bertocci, Claudia Giachini, Luciana Criscuoli, Elisabetta Micelli, Rita Picone

**Affiliations:** 1 Department of Biomedical, Experimental and Clinical Sciences “Mario Serio”, University of Florence, Florence, Italy; 2 Assisted Reproductive Technology Centre, Careggi University Hospital, Largo Brambilla 3, 50134 Florence, Italy; 3 University of Florence, Florence, Italy

**Keywords:** Egg donation, Oocyte vitrification, Cryo-bank, Infertility, Cumulative live birth, Assisted reproduction

## Abstract

**Purpose:**

To evaluate the effectiveness, efficiency, and safety of a transnational gamete donation (TGD) programme based on the shipping of vitrified donor oocytes.

**Methods:**

A retro-prospective observational study was conducted in the Assisted Reproductive Technology Center of the University Hospital of Florence, Italy. The study population included 622 consecutive donor oocyte cycles. A mean number of 6 vitrified oocytes per couple were shipped from two Spanish biobanks. In the receiving centre, gametes were warmed and inseminated and the subsequent embryo transfer (ET) was performed. The main outcome measurement was LBR. Secondary outcomes included oocyte survival rate, ICSI damage rate, normal fertilization, cleavage, and implantation rate (IR) in both ‘fresh’ and cryotransfer cycles.

**Results:**

A total number of 3536 mature oocytes were warmed with 81.4% surviving oocytes. 2PN oocytes were 1941 with an ICSI normal fertilization rate of 70.4% and a cleavage rate of 93.4%; 857 day-3 embryos were transferred in 498 women, 63 blastocysts in 44. Couples with at least one vitrified embryo were 181 (32.3%). IR was 25.1% and 33.1% for day-3 ET and blastocyst stage respectively. Crude pregnancy rate and LBR after the first ET were 35.5% and 27% correspondingly with a conservative cumulative LBR of 34% and an optimal LBR of 51.4%.

**Conclusion:**

Imported vitrified donor oocytes retain their competence and are capable of resulting in ongoing pregnancies and healthy babies in a proportion comparable to other existing systems as egg donation with vitrification/warming in the same laboratory and transnational fresh oocyte donation.

**Electronic supplementary material:**

The online version of this article (10.1007/s10815-020-01961-w) contains supplementary material, which is available to authorized users.

## Introduction

The most recent reports on Assisted Reproductive Techniques (ART) show a continuing growth of the cycles worldwide. At the same time, due to an ever-rising prevalence of age-related infertility and the introduction of egg banking, the proportion of cycles with egg donation (ED) is continuing to increase and is likely to increase further [1].

Data collected from the 19th annual report of the European in vitro fertilization (IVF) monitoring consortium sponsored by the European Society of Human Reproduction and Embryology (ESHRE), containing the data on ART cycles of 2015, reported an increment of 14.1% in ED treatments, compared with 2014 [2]. In the USA, ED cycles increased by 125% in a period of 16 years, from 10,801 in the year 2000 to 24,300 in 2016 [3].

In Italy, 10 years after Law 40/2004, the Constitutional Court (April 2014, the Court n. 162/2014) declared as unconstitutional the ban on heterologous assisted reproduction, thus legitimising egg and sperm donation to heterosexual intended parents.

The Careggi University Hospital in Florence became the first public health centre in Italy to offer heterologous ART, in the national health system. There was a sudden high demand to this hospital from couples coming from all over Italy, which could not be fulfilled. The only way to tackle the huge need of donor gametes was to rely on the Spanish biobanks.

In this context, the new model of Transnational Gamete Donation (TGD) was applied. It is based on the shipping of imported donor gametes (vitrified oocytes and frozen spermatozoa) from external biobanks, which were fertilised in the IVF laboratory of the receiving centre.

Egg banking has been possible as a result of the high success rates provided by vitrification. Oocyte vitrification efficiency was first demonstrated in ED programmes by a randomised clinical trial comparing the outcomes achieved using fresh versus vitrified oocytes from a unique cohort of donors and inseminated with the same semen sample [4]. In 2012, on the basis of the evidence provided by other randomised studies [5], the American Society for Reproductive Medicine declared that oocyte vitrification should no longer be considered an experimental procedure [6]. The consistency of vitrification results was further confirmed by a large study including almost 3500 ovum donation cycles [7]. Similarly, in 2017, Rienzi et al. demonstrated that oocyte vitrification provides high survival rates after warming, and similar pregnancy and live birth rates (LBR), compared with cycles with fresh oocytes [8].

The use of biobanks, in addition to the definite benefit of the immediate availability of donor oocytes, has further advantages in terms of the synchronisation between donors and recipients and the temporary quarantine of gametes in testing the donors for transmissible diseases [7].

The objective of this study was to describe the first experience of a TGD programme based on the transnational shipping of donor gametes (vitrified oocytes and cryo-spermatozoa) from biobanks to IVF labs. Ovum donation programme was analysed in terms of ART laboratory key performance indicators including a rigorous follow-up to report data on cumulative live birth rate (CLBR) and the safety of the babies born.

This study allowed us to critically evaluate the availability, effectiveness, efficiency, and safety of the TGD workflow over a 4-year period.

## Materials and methods

This retro-prospective observational study was conducted on infertile couples who attended the Centre for Assisted Reproduction of the University Hospital of Careggi, Florence, Italy. The study was approved by the Regional Ethical Committee (29th November 2016, CEAVC 10189, Amendment 16th May 2018 2018-017 CINECA 10189).

### Study population

The study population included consecutive donor oocyte IVF cycles conducted from January 2015 to December 2018. During the first visit, a detailed anamnesis was taken, focussing not only on the reproductive aspects but also on the pre-conception care of women. Our investigation was finalised to reduce the risk factors for adverse reproductive outcomes (obesity, smoking, etc.).

Couples were classified into women with hypergonadotropic hypogonadism; women at an advanced reproductive age, but still at a potentially fertile age; women with diminished ovarian reserve after failure of homologous fertilisation; women who know they are affected or have a significant genetic defect or a family history of a condition, for which the carrier status cannot be determined; women with poor quality of oocytes and/or embryos or repeated failed attempts at conception using ART; and women with iatrogenic infertility factor [9].

An informed consent containing information on the procedures, including details of egg banks, medical risk, and legal aspects, was signed by the couples before the ART cycle.

### Vitrification, shipping, and warming procedures

Following a European call for expressions of interest, two Spanish biobanks were selected for the procurement of donor oocytes. Oocyte donors were anonymous, voluntary, healthy women aged 20–35 years. Donor clinical evaluation comprises a thorough medical-gynaecological history, a physical examination, and an assessment for heritable diseases. Additionally, testing for sexually transmitted infections (STIs) and genetic screening (peripheral blood karyotyping, *Fragile X* premutation carrier, *CFTR* mutations, including the 5T allele) were performed [9].

The matching process considered the main phenotypic characteristics between the donor and recipient couple [9], including race or ethnicity, blood type, and Rhesus factor.

Each couple received six mature oocytes. Limited to the first period of the process organisation, the number of imported oocytes was 8–9. In selected cases, based on the age of the recipient and/or on couple’s specific needs, the request was limited to three oocytes.

Oocytes from the donor bank were vitrified with the Cryotop method for oocyte vitrification. All of the materials for vitrification were obtained from Kitazato (Kitazato, Shizuoka, Japan). Vitrification Kitazato® protocol with storage in Cryotop® open system was used to vitrificate oocytes by the donor bank. The process of oocyte vitrification consists of two stages. Firstly, oocytes are exposed to an equilibration process in which media are added sequentially during the incubation (12–15 min). In the second stage, oocytes are exposed to the vitrification solution by a two-step incubation. Oocytes are then placed on the Cryotop surface with the less vitrification solution as possible, and finally they are plunged quickly in liquid nitrogen (this step must be done by 1 min). Cryotops loaded with a maximum of three oocytes are covered with the protective caps and are stored in the oocyte bank.

The oocytes were transported from Spain to Italy in two ways: for the first 3 months in nitrogen vapours by plane and then by road in nitrogen liquid. After a few months, road transport by courier was replaced by the egg bank through door-to-door delivery. The latter way of shipping is still in use and is organised by the same biobanks through a team of specially trained IVF couriers.

In all cases, in order to monitor the temperature during the transit, the dry shippers for oocyte shipping were equipped with temperature probes and data logger.

After receiving the samples, the dry shipper was opened and re-filled with liquid nitrogen. Oocytes were moved from the dry shipper to a Styrofoam box to enable the identification of the cryo-carriers and then to a cryo-tank, for a temporary storage before warming.

Warming procedures were performed using the Kitazato Warming Media (Kitazato, Japan) according to the Cryotop® method described by Kuwayama et al. [10] and to the manufacturers’ instructions.

The protective cover was removed from the Cryotop while it was still plunged into liquid nitrogen, and the polypropylene strip of the Cryotop was plunged directly into 4 mL of thawing solution containing 1.0 M sucrose at 37 °C for 1 min. Subsequently, the oocytes were incubated at room temperature for 3 min in 300 μL of dilution solution 0.5 M sucrose and then two washes were performed in 300 μL of washing solution for 5and 1 min, respectively [10].

After warming, oocytes were incubated in fertilization medium (ORIGIO® Sequential Fert TM) at 6% CO_2_ and 5% O_2_ at 37 °C for 1.5–2 h; surviving oocytes were sperm-injected, placed in a dish with cleavage medium (ORIGIO® Sequential Cleav TM) under mineral oil (OVOIL™ Vitrolife), and incubated until day 3 [11].

After warming, oocytes were considered to be surviving when showing no dark/degenerated or contracted ooplasma and no cracked zona pellucida [12].

ICSI was performed using partner’s or donor’s sperm. Oocytes were assessed for fertilisation after 16–18 h, and those showing two pronuclei were cultured further until day 3. Embryo quality was assessed on days 2, 3, and at the blastocyst stage. The medium was changed on day 3 when embryos were cultured to day 5 (ORIGIO® Sequential Blast TM). Supernumerary embryos were vitrified on day 3 or day 5, using the Cryotop method for embryo vitrification [13, 14].

The definition adopted for the ICSI normal fertilisation rate was the proportion of injected oocytes with 2PN the day after injection. The ICSI damage rate was the proportion of damaged oocytes during the ICSI injection and included also degenerated oocytes by the time of fertilisation evaluation. ICSI normal fertilisation rate as the number of fertilized oocytes was assessed 17 ± 1 h post injection (presence of 2PN and 2PB). Cleavage rate was defined as the proportion of cleaved embryos on day 2 in relation to the number of 2PN/2PB oocytes on day 1 [15].

### Endometrial preparation of recipients

The programmed hormone replacement regimen consisted of an oral contraceptive pill (OCP) pre-treatment. After menses, all recipients were administered oral estradiol valerate (EV) (Progynova®, Bayer, Milan): 2 mg/day for 5 days, 4 mg/day for 4 days, and 6 mg/day on day 11 until ET. Women with functioning ovaries were downregulated with a single depot dose of a GnRH agonist (triptorelin) (Decapeptyl® 3.75; Ipsen Spa, Milan, Italy) 5 days before the interruption of the OCP. Approximately 11–12 days after initiating EV, patients underwent an endometrium evaluation by transvaginal ultrasound and serum estradiol (E2)/progesterone measurements. In case of poor response, transdermal EV at a dose of 100 μg once every 2 days was added (Estraderm TTS®, Novartis Farma, Origgio, Varese, Italy).

Once a triple layer endometrium reaching at least 7 mm and E2 levels > 150 pg/mL were observed, progesterone supplementation with 400 mg intravaginal capsules (Progeffik®/Prometrium®) every 12 h was started the same day of egg warming. The therapy (6 mg EV and 800 mg progesterone) was continued until the 10th week of gestation in case of pregnancy.

ET was performed with the use of ultrasound guidance and an ET catheter (Guardia Access K-JETS-7019; Cook) on days 2–3 or at the blastocyst stage. The guide catheter was introduced into the uterine cervix until it just passed the internal cervical os; then, the inner delivery catheter, previously loaded with embryos, was introduced into the uterine cavity with the catheter tip no closer than 15 mm to the fundus. One or two embryos were transferred into the uterine cavity. In the case of women over 45, presence of large and/or multiple myomas, previous uterine surgery, congenital uterine anomalies, Turner syndrome, and comorbidities, a single embryo was always transferred.

### Clinical outcome and pregnancy follow-up

Pregnancy was assessed through plasma β-hCG values 14 days after ET. The test was considered to be positive when hCG was > 10 mIU/mL. Clinical pregnancy (CP) was verified with the use of ultrasound at 6 weeks of gestational age. The presence of an intrauterine gestational sac confirmed a CP. The number of gestational sacs and heartbeats was recorded. Multiple gestational sacs were counted as one CP.

Implantation rate (IR) was defined as the number of gestational sacs divided by the number of transferred embryos; ‘CP’ as the presence of a gestational sac, with or without a fetal heartbeat, on ultrasonography; ‘ongoing pregnancy’ as a pregnancy beyond 12-week gestation; and ‘live birth’ as the delivery of one or more living infants [14, 16].

A pregnancy follow-up was conducted by two dedicated midwives by telephone every 3 months and continued up to 6 months post-partum. Information regarding the obstetric outcomes, mode of delivery, and well-being of the newborn infants were collected. When deemed necessary, patients were asked to send reports regarding pregnancy or childbirth via FAX or email. For patients delivering in our hospital, information was also verified through prenatal and delivery records.

Congenital malformations were defined as anomalies of development in a body structure of prenatal origin, potentially impacting an infant’s health, development, and/or survival [17].

### Statistical analysis

Statistical analysis was performed using Statistical Package for Social Science (SPSS) 18 (SPSS, 2009). Two-sided *P* values 0.05 were considered statistically significant in all the analyses.

Descriptive statistics were calculated for patient and treatment characteristics. Categorical data were expressed as number and percentage, while the quantitative variables were recorded as median, interquartile range (IQR), or mean ± standard deviation (SD) according to the data distribution. The statistical analysis compared the categorical variables with the *χ*^2^ test or the Fisher exact test.

The main outcome measurement was LBR. Secondary outcome measures included oocyte survival, ICSI damage, normal fertilisation, cleavage, implantation, and CP rates in both ‘fresh’ and cryo-transfer cycles.

LBR was expressed per ET episode. CLBR was calculated per warming cycle. The conditional LBR was defined as the number of live births at a specific cycle divided by the number of women receiving the treatment. CLBR incorporates fresh as well as thawed frozen ET [18]. Once a woman achieves her first live born baby from the treatment, she does not contribute any further to the cumulative rates. The conservative estimate of the CLBR corresponds to the number of live births up to and including a specific cycle, divided by the number of patients who ever received that treatment; the optimal estimate of the CLBR was based on the Kaplan–Meier estimates. A complete cycle was defined as all fresh and vitrified-warmed ET attempts resulting from one episode of donated oocyte warming [16, 19].

## Results

From January 2015 to December 2018, 561 couples were treated with ED for a total of 622 cycles (mean age 42.2 ± 3.8 years, 27–51 years). Table 1 shows the characteristics of the patients undergoing treatment (Table 1). The mean age of the oocyte donors was 25 ± 6 years.

**Table 1:** Patient characteristics table. Baseline characteristics of women and main indication at the beginning of the first cycle. Values are expressed as mean ± SD or percentages

	Num/mean ± SD	%
Number of treated couples	561	na
Female age (years)	42.2 ± 3.8 (range 27–51)	na
≤30 years	6	1.1
31–35 years	27	4.8
36–40 years	97	17.3
41–45 years	327	58.3
46–50 years	102	18.2
≥ 51 years	2	0.4
BMI	22.7 ± 3.1	na
Main indication		
Diminished ovarian reserve after failure of homologous fertilization	177	31.6
Advanced reproductive age	167	29.8
Hypergonadotropic hypogonadism	109	19.4
Poor-quality oocytes and/or embryos or repeated failed attempts	84	15.0
Genetic defect	17	3
Iatrogenic infertility	7	1.2

*SD*, standard deviation; *na*, not applicable

Couples undergoing the first cycle of ED were 561; in 60 couples, a second cycle was repeated, while only one couple had a third cycle.

A total number of 3536 mature oocytes were warmed for 561 cycles of ED (mean 6.3 ± 0.7 for patients, range 3–9) with 2878 (81.4%) surviving oocytes *(*5.1 ± 1.5 per patients). In 6 (1.1%) cycles, no oocytes survived for the treatment.

Overall, 2878 oocytes were injected by ICSI with a damage rate of 4.3% (123/2878), lower than the benchmark value of 5% [15]. In 6 cycles (1.1%), no fertilisation was observed. The number of oocytes with 2PN was 1941 (mean per couple 3.5 ± 1.5), with an ICSI normal fertilisation rate of 70.4% (mean for individual cycle 71.0 ± 23.7%). The number of cleaved embryos on day 2 was 1813, with a resulting cleavage rate of 93.4%. Both ICSI normal fertilization rate and cleavage rate were satisfactory results when compared with the corresponding value of competence of 65% and 95% [15].

**Fig. 1:**
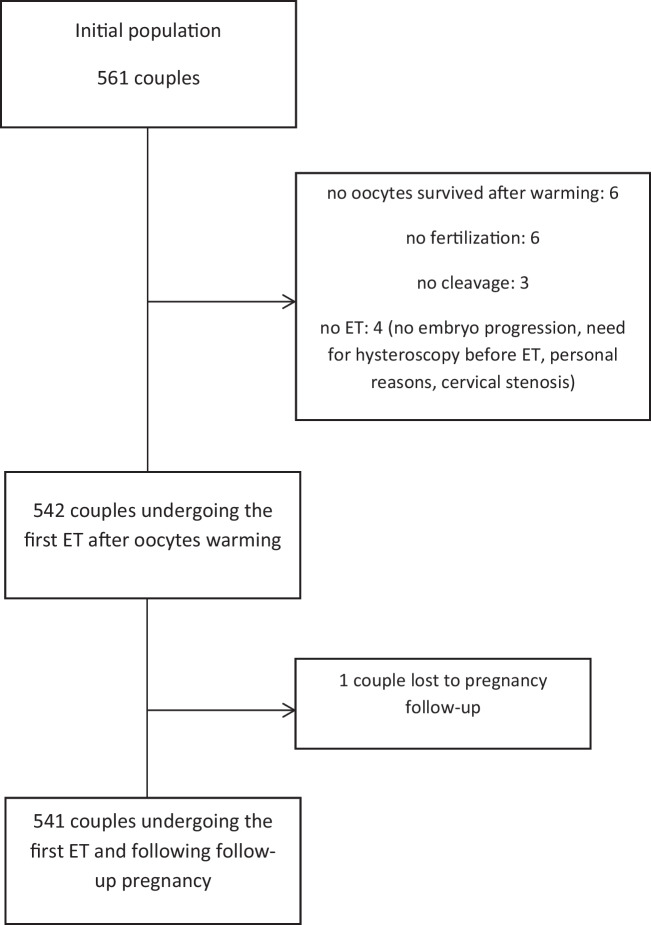
Flow chart of population study

Five hundred forty-two women underwent a ‘fresh’ ET (Fig. 1). The number of transferred day-3 embryos was 857 in 498 women (mean number for cycle 1.5 ± 0.7), while 63 embryos were transferred at the blastocyst stage in 44 cycles (mean 1.4 ± 0.5).

In 4 women, ET was not immediately performed. One hundred eighty-one (32.3%) couples had at least one vitrified embryo available for a further ET. A total of 299 embryos were vitrified for 177 couples (139 day-3 for 70 couples and 160 blastocysts were for 107 couples). In 4 cases, both day-3 embryos and blastocysts were vitrified.

As presented in Table 2, during the first ET, performed after the fertilisation of warmed oocytes, the proportion of cycles with day-3 ET was significantly higher than in the second ET, conducted with warmed embryos (91.7% versus 34.7%, *P* < 0.0001) (Table 2).

**Table 2:** Clinical outcome in all patients undergoing ET. The first cycle following oocyte warming and ETs

	First cycle							
	1st ET		2nd ET		3rd ET		4th ET	
	Num (%)	IC	Num (%)	IC	Num (%)	IC	Num (%)	IC
Num. of patients ET	541^§^	–	95 (17.6%)	–	14 (2.6%)	–	2 (0.8%)	–
Num. of embryos transferred, median (IQR)	2 (1.2)	–	1 (1.2)	–	1 (1.1)	**–**	2	**–**
Total [patients (embryos)]	541 (918)	–	95 (114)	–	14 (16)	**–**	2	**–**
Day-3 embryos [patients (embryos)]	497, 91.7% (855)	–	33, 34.7% (47)	–	7, 50% (8)	**–**	1 (1)	**–**
Blastocysts [patients (embryos)]	44.8.1% (63)		62, 65.2% (67)		7, 50% (8)		1(1)	
Clinical pregnancy (GS)	192 *, ** (35.5%)	31.4–39.7	31* (32.6%)	23.4–43.0	3 ** (21.4%)	4.7–50.8	1 (50%)	1.3–98.7
*1 GS*	147 (76.6%)	–	29 (93.5%)	–	3 (100%)	**–**	1 (100%)	–
*2 GS*	44 (22.9%)	–	1 (3.2%)	–	*–*	**–**		**–**
*3 GS*	1 (0.5%)	–	1 (3.2%)	–	*–*	**–**		**–**
Miscarriages	46 (23.9%)	18.1–30.6	7 (22.6%)	9.6–41.1	2 (66.7)	9.4–99.2	**–**	**–**
Extrauterine pregnancy	1 (/192, 0.5%)	–	–	–	–	**–**		–
Vanishing twin	5 (5/45, 11.4%)	–	1, 50%	–	–	**–**		**–**
Live birth	146 (27%)	23.3–30.9	25 (26.3%)	17.8–36.3	1 (7.1%)	0.2–33.9	1 (50%)	1.3–98.7
*Single*	110 (75.3%)	–	23 (92%)	–	1 (100%)	**–**	1	–
*Twin*	35 (24%)	–	2 (8%)	–	–	**–**		**–**
*Triplet*	1 (0.7%)	–	*–*	–	*–*	**–**		**–**
Newborns	183	–	27	–	1	**–**	1	**–**

*ET*, embryo transfer; *1st ET*, embryo transfer conducted with embryos developed from vitrified oocytes; *2nd ET and 3rd ET*, embryo transfers conducted with warmed embryos. ^§^One patient lost at follow-up; *P value equals 0.6731; **P value equals 0.4211

Overall, the pregnancy and delivery rates for the first oocyte warming procedure were 40.5% (95% CI, 36.4–44.5) and 30.8% (95% CI, 27–34.5) per donation cycle (561 women) and 42% (95% CI, 37.8–46.2) and 32% (95% CI, 28.1–36.1) per ETs (541 women). Crude PR and LB after the first ET were 35.5% and 27% respectively. In 95 couples, a second ET was performed, resulting in a 32.6% PR and 26.3% LBR, similar to the first ET (*P* < 0.05). In 14 patients, undergoing a 3rd and 4th ET, 2 further deliveries were observed (Table 2).

Given the low number of patients who underwent the third and fourth cycle, the results obtained were not comparable to the first two cycles. However, it was noteworthy that altogether two additional thaws added two live births (Table 2).

Table 3 shows the clinical data for 56 patients undergoing a second oocyte warming procedure. In these patients, we observed a clinical PR and a LBR of 32.1% and 21.4%, similar to the results obtained after the first cycle (*P* = 0.7246 and *P* = 0.4602 respectively) (Table 3).

**Table 3:** Clinical outcome in all patients undergoing ET in the second cycle of egg donation

Second cycle		
	1st ET	2nd ET
Num. of patients’ ET	56	4 (7.1%)
Num. of embryos transferred, median (IQR) patients (embryos)	2 (1.2)	
Total	56 (94)	
Day 3	53 (89)	
Blastocyst	3 (5)	
Clinical pregnancy (GS)	18 (32.1%)	1 (25%)
1 GS	17 (94.4%)	1 (100%)
2 GS	1 (5.6%)	–
3 GS	*–*	–
Miscarriages	6 (33.3%)	–
Extrauterine pregnancy	1 (/19, 5.3%)	–
Vanishing twin	–	–
Live birth	12 (21.4%)	1 (100%)
Single	11 (91.7%)	1
Twin	1 (9.1%)	–
Triplet		–
Newborns	13	1

Supplemental Table 1 shows pregnancy, implantation, and delivery rates according to the number of transferred embryos and corresponding embryo developmental stage (day-3 or blastocyst stage). Overall, 911 day-3 embryos were transferred in a total of 538 day-3 ETs; 139 blastocysts were transferred in 114 ETs (Supplemental Table 1).

IR was 25.1% and 33.1% for day-3 ET and blastocyst stage respectively (*P* = 0.0469). Although not significant, an increased IR for blastocyst stage transfer was observed in both cases of embryos developed from vitrified oocytes and day-3/blastocyst warming cycles (25.1% for day-3 ET and 36.5% for the blastocysts in the case of embryos developed from vitrified oocytes and 25.5% for day-3 ET and 32.8% for the blastocysts in the case of day-3/blastocyst warming cycles, *P* = 0.5279). IR was comparable with competency values of 25% for day-3 embryos and 35% for blastocyst stage, mainly for embryos developed from vitrified oocytes [20].

Among 538 patients with a transfer of 911 day-3 embryos, 185 (34.4%) pregnancies and 142 (26.4%) deliveries were observed. Similarly, in 114 patients with a transfer of 139 blastocysts, we observed 41 (36%) pregnancies and 31 (27.2%) deliveries (*P* = 0.8311 for PR and 0.9532 for LBR). We also analysed PR achieved after ET of embryos and blastocysts developed from vitrified oocytes (35% and 40.9% respectively) and PR in embryo and blastocyst warming cycles (27.3% and 35.5% respectively). No statistical significance was observed either while comparing pregnancies obtained after day-3 embryos and blastocyst ET (35% versus 40.9%, *P* = 0.5356 and 27.3% versus 35.5%, *P* = 0.5599), in both cases, or when comparing pregnancy rate after ET of embryos and blastocysts developed from vitrified oocytes versus corresponding warming cycles (35% versus 27.3 *P* = 0.4739 and 40.9% versus 35.5% *P* = 0.7155).

When day-3 embryos developed from vitrified oocytes were transferred, the live birth per ET was 26.6% with 25% of multiple pregnancies. In the case of ET involving blastocysts, live birth of 31.8% was observed with 21.4% of multiple pregnancies (*P* = 0.5646 for LBR and 1 for multiple pregnancies).

Among 324 patients with a transfer of two day-3 embryos developed from vitrified oocytes, we observed 28 (8.6%) multiple pregnancies. A higher percentage of multiple pregnancies was observed in 19 patients undergoing a transfer of two blastocysts (3/19, 15.8%; *P* = 0.3975).

In order to calculate the cumulative LBR, we took into account only patients undergoing all fresh and warmed ET resulting from one episode of oocyte warming. Thus, women with vitrified embryos which had not been warmed by the end of our study were excluded from cumulative live birth analyses. In 506 patients who completed their cycle, we observed conservative CLBR of 34% (Table 4).

**Table 4:** Live birth rates per complete cycle and cumulative live birth rates per woman

		Num. of women	Num. of women with at least one live birth	Conditional live birth rate (%)	95% CI	Conservative cumulative live birth (%)	95% CI
Cycle 1	1st ET	506	146	28.8	24.9–33.0	28.8	24.9–33.0
	2nd ET	86	25	29.1	19.8–39.9	33.8	29.7–38.1
	3rd ET	12	1	8.3	0.2–38.5	34.0	29.9–38.3

**Fig. 2:**
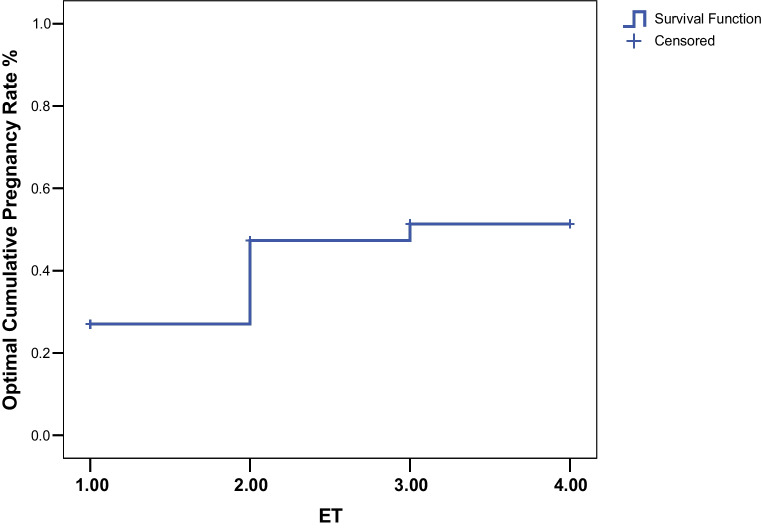
Optimal cumulative live birth rates per woman over consecutive ETs following the first cycle of warming oocytes. The optimal estimate of the cumulative live birth rate assumed that the live birth rate among women who did not return for further treatment would be the same as the rate among those who continued treatment. ET, embryo transfer episode

The optimal estimate of the cumulative live birth rate, calculated on 541 women, was 51.4% at the third ET (Fig. 2).

A total of 226 babies were born (212 after the first warming cycle and 14 after the second). In 3 cases, 1.3%, congenital malformations were diagnosed: one of them presented achondroplasia, the second with transposition of the great arteries (TGA), and the third with congenital hydronephrosis.

## Discussion

The model of TGD, also referred to as ‘two countries-two labs’, represents a form of collaborative embryo-lab process based on the shipping of vitrified oocytes from foreign biobanks and subsequent warming and fertilisation in the same centre performing ET.

In the present study, we observed a conservative CLBR of 34% and an optimal CLBR of 55.3%, demonstrating that imported vitrified donor oocytes retain their competence and are capable of resulting in ongoing pregnancies and healthy babies in a proportion comparable to other existing systems such as ED with vitrification/warming in the same laboratory [5] and transnational fresh oocyte donation (TOD) [21].

The survival of the warmed oocytes of 81.4% was lower than the 90.4% reported by Cobo et al. [5]. ICSI normal fertilisation rate (70.4%) and IR for both day-3 embryo and blastocyst (25.1% and 36.5% respectively in the case of embryos developed from vitrified oocytes) satisfied KPI competency value [15]. Cleavage rate of 93.4% was a little lower than the corresponding KPI competence value (95%), but resulted in data similar to that reported by studies conducted on vitrified donor oocytes [22].

The reduction in oocyte survival rate after warming might be related to the process of vitrification/warming or to the transnational shipping. At the same time, we cannot exclude attributing this result to the initial number of vitrified oocytes. In this study, the mean number of six oocytes was assigned to each couple. In the data by Cobo et al. [5], a minimum of eight oocytes per donation was required. We recognise that the survival of warmed oocytes is the first part and one of the most crucial aspects of the whole process of egg donation. A study is currently underway at our centre to clarify this aspect.

Our system results from the necessity to offer each patient the maximum chance of success in terms of LBR within the public health service, in accordance with the Italian law on assisted reproduction.

A fundamental objective in health systems is to determine the best use of the limited funds available to promote health and provide healthcare [23]. It is therefore important to *use* these *resources efficiently*. Furthermore, the Italian law (L40) does not allow the cryopreservation of embryos, except in strictly selected cases and, at the same time, prohibits the donation of embryos. The advanced age of our patients should also be taken into account. In fact, during the first warming cycle, more than 80% (82.5%, 463/561) of the women were over 40 and 26% (146/561) over 45 years. In this age group, patients will rarely seek a second pregnancy. For all these reasons, the total number of vitrified embryos must be kept to the minimum. The number of six oocytes required from the biobanks was considered the best compromise in terms of cost-effectiveness. Furthermore, after evaluating the results achieved by our centre in women over 44, it seemed appropriate to reduce the number of oocytes required to 3–4, in this age group.

The optimal day of ET, cleavage stage on day 3 versus blastocyst transfer on day 5, remains controversial [24–28]. Several authors reported improved LBR in fresh IVF-ET cycles with blastocyst in comparison to day 3 ET [29], while others still debate whether ET at the blastocyst stage is superior to the early cleavage stage [26]. Even less data are available about the ideal embryo developmental stage of ET in the cases of embryos obtained from vitrified donor oocytes [30].

In the present study, embryos obtained after warming of donor oocytes were mainly transferred on day 3 (91.7% of cycles) while in cases of cryo-transfers, blastocysts were mostly transferred (65.2% of cycles). Blastocyst ET showed a *trend toward a higher implantation* and LB rates compared with day-3 ET cycles (implantation rate: 25.6% in single day-3 ET versus 40% single blastocyst ET, *P* = 0.2114; LBR: 18.6% single day-3 ET versus single blastocyst ET, *P* = 0.7165). Patients undergoing the transfer of one or two day-3 embryo/s obtained a similar pregnancy rate, versus women who underwent ET of a single blastocyst (26.3% and 24% respectively). These results might support the strategy to transfer day-3 embryos in the setting of ED procedure, when embryos are obtained from vitrified donor oocytes.

Furthermore, *well*-*planned* and adequately *powered* studies on the optimal developmental stage of ET are certainly needed. Actually, our study population included an unselected group of patients (i.e. irrespective of the couples’ indication, woman’s age, embryo quality, and male factor). Conversely, reports published earlier suggested that there could be a paternal effect on embryo development [31]. Moreover, in our centre, we have a *single ET policy* (both day-3 and blastocyst stage) for couples undergoing ED cycles, identified as being at high risk for adverse obstetric outcomes (women over 45, presence of large and/or multiple myomas, previous uterine surgery, congenital uterine anomalies, Turner syndrome, and comorbidities). The same conditions may also account for lower implantation and LB rates in these patients [32–35].

The increase in twin pregnancies (44%) following the exceptional transfer of two blastocysts stressed the higher implantation potential of day-5 embryos. Since this data emerged, we carried out transfers of only single blastocyst.

Choosing the patient to transfer an embryo at the blastocyst stage remains a clinical challenge, as it is related to the possibility of having no embryos to transfer. Interestingly, some day-3 stage embryos that do not survive in extended in vitro culture may be rescued in the uterine environment [36, 37]. Racowsky et al*.* observed that lack of 8-cell embryos on day 3 resulted in no pregnancy for blastocyst transfers, versus PR of 33% for day-3 ET [37]. The donor’s age and AMH level, number of mature oocytes, embryo morphology, and number of 8-cell embryos on day 3 could be the determining factors for the optimal day of transfer. Additionally, suboptimal quality embryo culture might compromise extended in vitro culture [38].

Since there was no previous experience in the context of a ‘two countries-two centres’ reality, the number and the cleavage stage of embryos to be transferred in different age groups had to be verified first. Thus, in the beginning, we preferred to transfer day-3 embryos and eventually freeze embryos at the blastocyst stage. After observing the first results, our approach is changing and we prefer to transfer embryos at the blastocyst stage, when we get more than 3 embryos (data not included in the present study).

Results in terms of pregnancy rate and LBR after the first ET in the first cycle were comparable to the first ET of the second cycle (*P* < 0.05). Thus, the chance of having a live birth after an unsuccessful fresh cycle if they continue with further ART treatment remained similar. These results confirmed that vitrification at the early cleavage stage or day-5 stage of embryos obtained from the imported donor’s oocyte has no effect on implantation and delivery rate. In a retrospective cohort study conducted on a total of 471 warming cycles of 796 vitrified embryos developed from vitrified oocytes, Cobo et al. observed that double vitrification has no impact on delivery rates [30]. To the best of our knowledge, there are no previous studies investigating the effect of ET of vitrified embryos generated from imported donors’ vitrified oocytes.

The prevalence of congenital malformations (1.3%) observed in the present study was reassuring with regard to live births resulting after ED in a ‘two countries-two labs’ programme. The reported prevalence of major congenital malformations in different populations around the world has shown considerable variation and ranges from less than 1 to 8% [39–41]. The European Surveillance of Congenital Anomalies (EUROCAT) [42] recorded a total prevalence of major congenital anomalies of 256.03 per 10,000 births for the year 2017 (2.6%), 193.37 (1.9%) in respect of live births.

The strict collaboration between the two labs and the constant monitoring of its obtained results constituted crucial points for a successful programme. The exchange of information regarding the results and the training of the operators at the two centres are the bases of this cooperation and have led us to modify the process variables. The shipping method, the culture media, the vitrification/warming process, related embryologists’ competence/expertise, and improved ART laboratory technologies were significant factors. The constant monitoring of the process with the implementation of necessary corrections allowed us to progressively reach a steady state with satisfactory performances, as measured by KPIs*.*

The TGD system actually starts with the gametes transport from the site where the oocyte retrieval is performed to the site where oocytes are processed. Indeed, this phase can be very critical for the overall quality of the sample and the future reproductive outcomes. In a ‘two country-two lab’ system, egg bank door-to-door road delivery showed the way to guarantee the best transport of the gametes. This method allowed the biobanks to organise their own gamete transport through a team of specially trained IVF couriers, who have all the information in order to prevent any violation of temperature/pressure regime during the shipping, and avoid variables related to air transport.

Secondly, the sharing and standardisation of kits and protocols for vitrification have also proven to be of utmost importance. In order to eliminate inter-operator variability, two embryologists have been dedicated exclusively to this process at our centre.

Our data are difficult to compare with TOD’s method for a number of reasons. Firstly, in the TOD system, the entire process of fertilisation and vitrification of embryos is performed in the same centre where the donors submit to oocyte retrieval; secondly, it involves a double need for transporting seminal fluid first and embryos next, with logistical and safety implications.

In conclusion, to the best of our knowledge, this is the first retro-prospective report on ED with TGD as a *modus operandi* and showed the capacity to guarantee valid results in terms of effectiveness, safety, and costs.

The TGD system is based on the optimisation of the results, taking into account the number of oocytes and, consequently, the number of embryos vitrified, not only for reasons of compliance with the law in force but also for bioethical reasons.

Further studies are needed to confirm our results and to clarify unanswered questions, such as the variables affecting vitrified oocyte survival and optimal number and the cleavage stage at which it is preferable to transfer embryos in such a process.

### Electronic supplementary material

ESM 1 (DOCX 34 kb)
