# Correction to: ‘Two countries-two labs’: the transnational gamete donation (TGD) programme to support egg donation

**DOI:** 10.1007/s10815-023-03006-4

**Published:** 2024-01-03

**Authors:** Maria Elisabetta Coccia, Francesca Rizzello, Savio Wakunga, Laura Badolato, Paolo Evangelisti, Francesco Bertocci, Claudia Giachini, Luciana Criscuoli, Elisabetta Micelli, Rita Picone

**Affiliations:** 1 Department of Biomedical, Experimental and Clinical Sciences “Mario Serio”, University of Florence, Florence, Italy; 2 Assisted Reproductive Technology Centre, Careggi University Hospital, Largo Brambilla 3, 50134 Florence, Italy; 3 University of Florence, Florence, Italy

**Correction to: Journal of Assisted Reproduction and Genetics 37(12): 3039-304**

10.1007/s10815-020-01961-w

In this article the author’s name “Claudia Giachini” was incorrectly written as “Giachini Claudia”.

The original article has been corrected.

