# Supplementary material for: ‘Two countries-two labs’: the transnational gamete donation (TGD) programme to support egg donation
Source: J Assist Reprod Genet. 2020 Oct 12;37(12):3039–49. doi: 10.1007/s10815-020-01961-w (PMC7714895; doi:10.1007/s10815-020-01961-w)
Supplement: Supplementary file 1 — (DOCX 34 kb) [file 10815_2020_1961_MOESM1_ESM.docx]

Supplemental Table 1: Pregnancy, implantation and delivery rates according to the number of transferred embryos, embryo developmental stage (day-3 or blastocyst stage). GS= Gestational sacs, ET=Embryo transfer
